# NADP(H)-dependent biocatalysis without adding NADP(H)

**DOI:** 10.1073/pnas.2214123120

**Published:** 2022-12-27

**Authors:** Ryan A. Herold, Raphael Reinbold, Christopher J. Schofield, Fraser A. Armstrong

**Affiliations:** ^a^Department of Chemistry, University of Oxford, Oxford OX1 3QR, United Kingdom; ^b^Department of Chemistry and the Ineos Oxford Institute for Antimicrobial Research, University of Oxford, Oxford OX1 3QY, United Kingdom

**Keywords:** biocatalysis, NADPH, isocitrate dehydrogenase, electrocatalysis, nanoconfinement

## Abstract

Isocitrate dehydrogenase 1 (IDH1) naturally copurifies and crystallizes in a resting state with a molecule of its exchangeable cofactor, NADP^+^/NADPH, bound in each monomer of the homodimer. We report electrochemical studies with IDH1 that exploit this property to reveal the massive advantage of nanoconfinement to increase the efficiency of multistep enzyme-catalyzed cascade reactions. When coloaded with ferredoxin NADP^+^ reductase in a nanoporous conducting indium tin oxide film, IDH1 carries out the complete electrochemical oxidation of 6 mM isocitrate (in 4mL) to 2-oxoglutarate (2OG), using only the NADP(H) that copurified with IDH1 and was carried into the electrode pores as cargo—the system remains active for days. The entrapped cofactor, now quantifiable by cyclic voltammetry, undergoes ~160,000 turnovers during the process. The results from a variety of electrocatalysis experiments imply that the local concentrations of the two nanoconfined enzymes lie around the millimolar range. The combination of crowding and entrapment results in a 10^2^ to 10^3^-fold increase in the efficiency of NADP(H) redox cycling. The ability of the method to drive cascade catalysis in either direction (oxidation or reduction) and remove and replace substrates was exploited to study redox-state dependent differences in cofactor binding between wild-type IDH1 and the cancer-linked R132H variant that catalyzes the “gain of function” reduction of 2OG to 2-hydroxyglutarate instead of isocitrate oxidation. The combined results demonstrate the power of nanoconfinement for facilitating multistep enzyme catalysis (in this case energized and verified electrochemically) and reveal insights into the dynamic role of nicotinamide cofactors as redox (hydride) carriers.

## Significance

Within cells, enzymes and cofactors catalyzing multistep processes (cascades) are often confined together—either in enclosures (e.g., organelles) or via physical association (metabolons). Nanoconfinement, offering potential general advantages for catalysis, can also be achieved by loading enzymes and their exchangeable cofactors into a porous, electrically conducting inorganic material, thereby enabling catalysis to be channeled, energized, and investigated electrochemically. Such nanoconfinement enables a cascade comprising electroactive ferredoxin NADP^+^ reductase and isocitrate dehydrogenase to be active for days, catalyzing exhaustive oxidation of bulk isocitrate by recycling trapped NADP(H) carried in on isocitrate dehydrogenase. Nanoconfinement massively increases the efficiency of cofactor-dependent cascade catalysis and has conceptual relevance for prebiotic evolution where complex organic molecules might have formed in gaps and cracks of minerals.

Nanoconfinement is a strategy employed by biological systems to optimize the efficiency of many cellular processes including carbon fixation, respiration, and photosynthesis. Nanoconfinement strategies include both volumetric confinement (eukaryotic organelles/bacterial microcompartments) and colocalization of enzymes (metabolons) in a manner proposed to enable exclusion of competing enzymes/pathways, increase local enzyme concentrations, create concentration gradients/charge separation, and minimize escape of reactive (sometimes toxic) intermediates or exchangeable cofactors: the latter might thus be efficiently recycled (1–3). To understand and exploit these benefits, artificial systems have been developed to mimic biological nanoconfinement strategies (4–7).

**Scheme 1.**
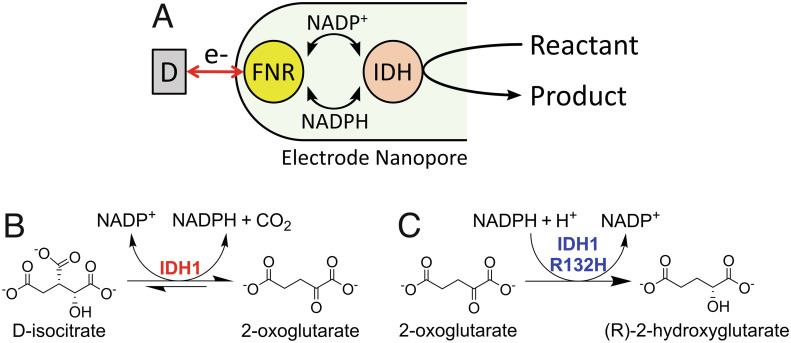
Electrochemical Leaf and wild-type and variant IDH1 reactions*.* (*A*) Principle of the Electrochemical Leaf: NADP(H) is recycled between FNR and an NADP(H)-dependent dehydrogenase; “D” represents the “dashboard,” the equipment used to control and monitor the reaction, which is composed of a potentiostat, a computer, and an electrode rotator. (*B*) The reversible wild-type IDH1 reaction. (*C*) The neomorphic reaction catalyzed by cancer-associated IDH1 variants, including IDH1 R132H. Molecules are shown as the forms predominating at pH = 7 to 8 (8).

The Electrochemical Leaf (e-Leaf) is a technology that mimics biological nanoconfinement by trapping enzymes in a nanoporous conducting metal oxide (MO) layer deposited at micron scale on a supporting electrode (8–12). The technology enables the user to energize, control, and observe complex enzyme cascade reactions in real time. As shown in Scheme 1A, the e-Leaf exploits nanoconfinement in a network of pores (5 to 100 nm in diameter) (11) to enable rapid recycling of the transferable, hydride-carrying nicotinamide cofactor [NAD(P)(H)] between ferredoxin-NADP^+^ reductase (FNR), an electroactive flavoenzyme, and members of the superfamily of NAD(P)(H)-dependent dehydrogenases (13) including isocitrate dehydrogenase 1 (IDH1) (8). The electrode is represented in shorthand as (E1 + E2)@MO/support, where E1 = FNR, E2 = NADP(H)-dependent dehydrogenase. The rate of an enzyme cascade can be observed directly and in real time because FNR acts as a transducer by performing rapid electron exchange between the electrode and the active site flavin and either oxidizing NADPH or reducing NADP^+^ depending on the electrode potential applied. The transferable NADP(H)-cofactor is efficiently recycled by the dehydrogenase, and the overall turnover rate is recorded as a current. A feature made possible by the tight coupling achieved between electron and hydride (cofactor) transfer is that the NADP(H)-dependent dehydrogenase is itself rendered “electroactive” (8). This “electrification” of cascades can be extended to include enzymes of other classes—E3, E4, etc that connect to the dehydrogenase via preceding or subsequent reactions of its substrates or products (9).

Human IDH1 is a Mg^2+^-dependent cytosol-localized enzyme that catalyzes the reversible, oxidative decarboxylation of *D*-isocitrate to 2-oxoglutarate (2OG) and carbon dioxide using NADP^+^ (Scheme 1*B*) (14). Together with its mitochondria-localized isoform, IDH2, the IDH-encoding genes are the most frequently mutated metabolic genes associated with cancer (15, 16), with active site substitutions in IDH1 and IDH2 resulting in a gain of function (“neomorphic”) activity: i.e., catalysis of the reduction of 2OG to the oncometabolite 2-hydroxyglutarate (2HG) using NADPH (Scheme 1*C*) with a decrease in isocitrate oxidation efficiency (17). In the case of IDH1, the focus of this study, the most common substitutions occur at arginine-132, with the most common cancer-associated substitutes being histidine (R132H) and cysteine (R132C) (18).

A characteristic of both recombinant wild-type IDH1 and the cancer-associated IDH1 R132H variant is that they copurify as dimers with one molecule of NADP(H) tightly bound at each monomer active site (*SI Appendix*, Fig. S1 *A* and *B*) (14, 19–22). Other proteins that copurify with bound NAD(H) (23–32) and NADP(H) (33–39) include the nicotinoproteins (28–32, 37, 38, 40) that apparently use NAD(P)(H) as a permanently bound prosthetic group (instead of an exchangeable cofactor) to perform a variety of reactions. The limited examples of NAD(P)(H)-dependent dehydrogenases copurifying with nicotinamide cofactors (23, 24, 32–35) suggest that extremely tight cofactor binding may be unusual.

Such a high binding affinity for nicotinamide may appear detrimental for enzymes that use it as an exchangeable cofactor, but it should be borne in mind that a copurified complex represents just one state among many, within or outside the catalytic cycle. Indeed, IDH1 enzymes do not have particularly low *K*_m_ values for NADP(H) (they lie in the range 10 to 100 µM) (20, 41) compared to other NAD(P)(H)-dependent dehydrogenases, suggesting that different conformations may vary in their affinities for NADP(H): accordingly, transient kinetic studies have shown that ~50% of enzyme-bound NADPH is released from wild-type IDH1 after it undergoes a conformational change induced by Mg^2+^ and isocitrate binding (14). The copurified NADP(H) remains bound even when large volume buffer exchanges are used (~12 million-fold dilution)—its release occurring only after Mg^2+^and substrate bind (14). Because the extremely tight binding of two molecules of NADP(H) is apparently limited to a “resting state” of the dimer enzyme, it can be carried into the electrode pores as a quantifiable cargo on each IDH1 molecule before being released on command. This property offered a special opportunity to carry out a quantitative study using the Electrochemical Leaf to investigate the benefits of nanoconfinement for an enzyme cascade, focusing on how efficiently NADP(H) is used by a pair of enzymes to which the cofactor is coupled, to advance both fundamental understanding and provide technological insight.

## Results and Discussion

### Wild-Type IDH1 and IDH1 R132H Copurify with Enzyme-Bound NADP(H).

**Fig. 1.**
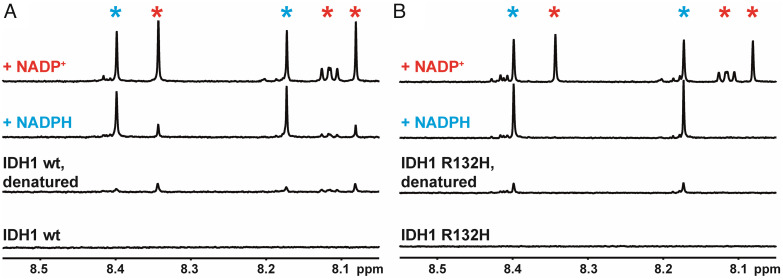
^1^H NMR data showing the release of copurified enzyme-bound NADP(H) from wild-type IDH1 (*A*) and IDH1 R132H (*B*) upon thermal denaturation (see *SI Appendix*, Fig. S3 for the equivalent experiment with FNR). Reading vertically from the lower spectra i) folded IDH1 protein—NADP(H) is initially enzyme-bound, hence not observed; ii) denaturation releases NADP(H) into solution. (*A*) Wild-type IDH1 copurifies with an approximately 2:1 mixture of NADP^+^ (red asterisk) and NADPH (blue asterisk). (*B*) IDH1 R132H apparently copurifies exclusively with NADPH.

To confirm that purified recombinant wild-type IDH1 and IDH1 R132H enzymes contained copurifying NADP(H), we performed NMR experiments (Fig. 1 *A* and *B*). In the case of IDH1 R132H (Fig. 1*B*), NADPH, but not NADP^+^, copurified with the enzyme (see later). By contrast, wild-type IDH1 (Fig. 1*A*) copurified with a mixture of NADP^+^ and NADPH bound, in a ratio of about 2:1 in favor of NADP^+^. Non-denaturing mass spectrometry analysis showed that dimeric wild-type IDH1 copurifies with an average NADP(H) active site occupancy of 46%, giving a stoichiometry of approximately NADP(H):IDH1_dimer_ = 1:1, while IDH1 R132H copurifies with approximately 97.5% NADP(H) occupancy (i.e., the NADP(H):IDH1 R132H_dimer_ ratio is close to 2:1) (*SI Appendix*, Fig. S2). These data are consistent with the amount of NADP(H) released by each enzyme following denaturation as quantified by ^1^H NMR (Fig. 1 *A* and *B*), although this method was less precise due to the low concentrations present. It was previously reported that both IDH1 enzymes copurify with roughly 90% NADP(H) active site occupancy (20). The different data concerning the NADP(H):IDH1 stoichiometry and whether wild-type IDH1 copurifies with a mixture of NADP^+^/NADPH (19) or exclusively NADPH (14, 20) possibly reflect differences in expression/purification methods, emphasizing the importance of verifying the status of IDH1-copurified NADP(H) under our expression and purification conditions. To confirm that FNR did not copurify with NADP(H), an analogous ^1^H NMR experiment was performed which also clearly showed the release of its prosthetic group, flavin adenine dinucleotide (FAD), upon denaturation (*SI Appendix*, Fig. S3).

### Copurified NADP(H) Is Sufficient for Stable Enzyme Catalysis under Nanoconfinement.

After establishing that both wild-type IDH1 and the R132H variant copurify with NADP(H) and that FNR does not, we performed electrochemical experiments to test if the copurifying NADP(H) alone is sufficient to sustain steady-state catalysis through NADP(H) recycling between IDH1 and FNR when coentrapped inside the nanopores of an indium tin oxide (ITO) electrode. Both enzymes were loaded together by pipetting a concentrated solution (4 to 5 µL) onto the electrode and incubating at room temperature for 30 to 45 min before rinsing thoroughly with buffer (only a tiny fraction of enzyme enters the nanopores while the rest is removed by rinsing: see *SI Appendix*, Fig. S5).

**Fig. 2.**
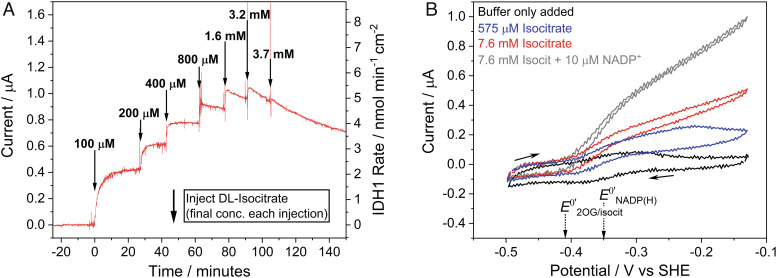
Electrochemical nanoconfinement experiments demonstrating that wild-type IDH1 activity is clearly observed using only IDH1-copurified NADP(H) (no NADP(H) was added). (*A*) Chronoamperogram showing IDH1 activity as increasing concentrations of DL-isocitrate are titrated into the solution. The injection concentrations shown are the final concentrations of each addition of isocitrate (not cumulative). (*B*) Cyclic voltammetry showing wild-type IDH1 activity at different concentrations of isocitrate. The gray trace shows IDH1 activity when 10 µM NADP^+^ was added to the solution. Conditions (*A* and *B*): (FNR+IDH1)@ITO/PGE electrode, temperature 25 °C, volume 4 mL, pH = 8 (100 mM HEPES, 10 mM MgCl_2_), and enzyme loading ratios (molar): FNR/IDH1; 2/1. (*A*): electrode area 0.06 cm^2^, 1,000 rpm, potential *E* (vs. standard hydrogen electrode, SHE) = +0.2 V. (*B*): 0.03 cm^2^ electrode area, scan rate 1 mV/s, stationary electrode. Racemic DL-isocitrate was used for both *A* and *B*.

Both wild-type IDH1 and IDH1 R132H showed strong catalytic responses when their respective substrates were titrated into the cell solution even though there was no NADP(H) added (Fig. 2 and *SI Appendix*, Fig. S6). The increases in current upon substrate addition are consistent with previous results using both enzymes in the e-Leaf (8). Neither isocitrate nor 2OG is electroactive alone within the range of electrode potential applied (8). Interestingly, the early additions of isocitrate to wild-type IDH1 (Fig. 2*A*) show a lag period that was not observed in subsequent isocitrate additions. The lag period indicates an activation process, perhaps as proposed by Roman et al. for IDH1 (14). The cyclic voltammetry results for wild-type IDH1 (Fig. 2*B*) reveal that low levels of isocitrate begin to deplete close to the electrode–solution interface (the blue trace in Fig. 2*B* shows that the oxidation current reaches a maximum level and then decreases). At high isocitrate levels, the catalytic rate is controlled by the rate at which FNR receives electrons to reduce the NADP^+^: the red trace shows that the current continues to increase with the electrode potential. The gray trace shows that the current increases when external NADP^+^ is added. An important observation is that the “buffer only added” voltammogram (black trace) in Fig. 2*B* is not that which would be expected for FNR alone; instead, the trace contains a component due to coupled NADP(H) cycling (there is a sharp reduction peak and a broader oxidation peak with a significant trailing tail). This observation was investigated in detail (see below) to quantify the IDH1 and NADP(H) present in the nanopores.

### IDH1-FNR Cascade Using Only IDH1-Copurified NADP(H) Is Highly Stable in the Electrode Nanopores.

**Fig. 3.**
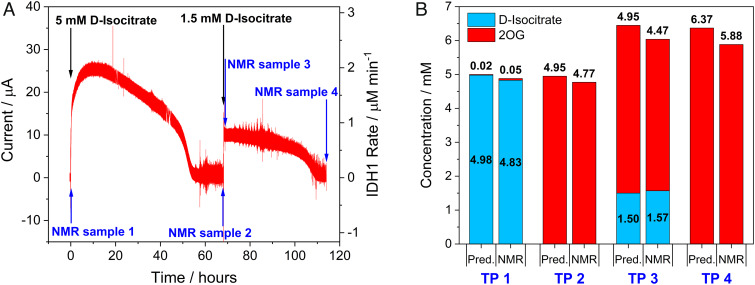
Scaled-up and time-extended wild-type IDH1 experiment (4 cm^2^ electrode, 4 mL stirred solution) showing quantitative conversion of 6 mM *D*-isocitrate to 2OG using only IDH1-copurified NADP(H). (*A*) Chronoamperogram showing IDH1 activity over 5 d. (*B*) Bar chart comparing predicted yields (based on charge passed) with ^1^H NMR-quantified substrate (*D*-isocitrate) and product (2OG) at each time point (TP). The NMR measurements correspond to samples taken from the working electrode solution, and the concentrations shown are corrected for the volumes of samples taken/injections made. Conditions for (*A*): (FNR + IDH1)@ITO/Ti foil electrode, area 4 cm^2^, solution agitated by stirring, temperature 25 °C, volume 4 mL, potential *E* (vs. SHE) = +0.2 V, pH = 8 (100 mM HEPES, 10 mM MgCl_2_). Enzyme loading ratios (molar): FNR/IDH1; 2/1. *Note: After completion of the electrochemical experiment, NMR analysis of the counter electrode solution showed that 0.78 µmol of 2OG and 2.6 µmol of isocitrate were present, having crossed through the glass frit from the working electrode chamber during the experiment. The “crossed-over” 2OG was added to the time point 4 NMR value to show the total product made. When the isocitrate in the counter electrode solution is included, the residual isocitrate (0.661 mM) + 2OG produced (5.88 mM) = 6.541 mM at time point 4, a value close to the total 6.5 mM of isocitrate initially added.

To investigate the stability of the electrocatalytic system using only copurifying, nanoconfined NADP(H), an experiment was conducted to quantify the products formed over a 5-d period (Fig. 3). The electrode surface area was increased 67-fold to 4 cm^2^ (double-sided 1 × 2 cm ITO on titanium foil), and the total amount of enzyme applied to the electrode was increased 4-fold (see *Materials and Methods*). Based on the total amount of enzyme loaded, approximately 2.4% of the FNR applied to the larger 4 cm^2^ electrode from the experiment in Fig. 3 was taken up by the electrode, compared to only ~0.6% in the loading of the 0.06-cm^2^ electrode. This difference likely resulted because the total amount of enzyme used was scaled-up only ~4-fold while the electrode surface area was 67-fold greater.

After 45 min of contact with the enzyme solution, the electrode was thoroughly rinsed with buffer and placed into an electrochemical cell containing 4 mL buffer solution with 10 mM MgCl_2_. A baseline current (0 A) was recorded, then enantiopure *D*-isocitrate was injected into the cell to give a concentration of 5 mM, and a sample was taken for NMR analysis shortly thereafter at time point 1 (TP 1, Fig. 2*B*). The current increased to a maximum of approximately 26 µA before decreasing slowly over the next 2 d, eventually reaching zero after 55 h: at this point, all the isocitrate had been converted to 2OG based on the charge passed (Fig. 3). At TP 2 (Fig. 3*B*) i.e. at 68 h, a sample was taken for NMR analysis to quantify the amount of 2OG produced, and a further 1.5 mM *D*-isocitrate was added (TP 3, Fig. 3*B*) resulting in an increase in the current. The reaction was allowed to continue until nearly all of the isocitrate had been converted based on the measurement of charge passed (TP 4, Fig. 3*B*); a further sample was then taken for NMR analysis (Fig. 3).

### Quantifying FNR, NADP(H), and IDH1 in the Electrode Nanopores.

The FNR that is adsorbed and electroactive in the electrode nanopores can be quantified using cyclic voltammetry by integrating the area under each nonturnover redox peak resulting from fast two-electron transfer to/from the FAD prosthetic group (8, 42). For example, based on eight 0.06 cm^2^ electrodes loaded with FNR and wild-type IDH1 (2:1 molar ratio), the average FNR loading was 161 ± 19 pmol cm^−2^ (±SD). Until now, however, no method has been available to allow the quantification of other enzymes trapped in the nanopores along with FNR because they do not perform long-range electron transfer: instead, a qualitative relationship between the different enzymes in the e-Leaf has been adopted, relying on optimizing the loading ratios to achieve the desired electrocatalytic performance (8, 9). As explained next, the fact that IDH1 enters the electrode nanopores with copurifying NADP(H) provided a special opportunity to measure the amounts of entrapped IDH1 and NADP(H) in the addition to FNR.

The “buffer only added” 1 mV/s voltammogram (Fig. 2*B*) recorded for the electrode coloaded with FNR and IDH1 exhibits a waveform that is not characteristic of FNR electron exchange alone. Although NADP(H) is bound tightly by IDH1 in its substrate-free inactive state, the IDH1·NADP(H) complex exists in dynamic equilibrium, a property which has been exploited by Roman et al. to study the IDH1·NADP^+^ complex in the open inactive conformation: old yellow enzyme (NADPH dehydrogenase 2) incubated alongside wild-type IDH1 was able to oxidize transiently released NADPH to NADP^+^, which was then reacquired by IDH1 (14).

**Fig. 4.**
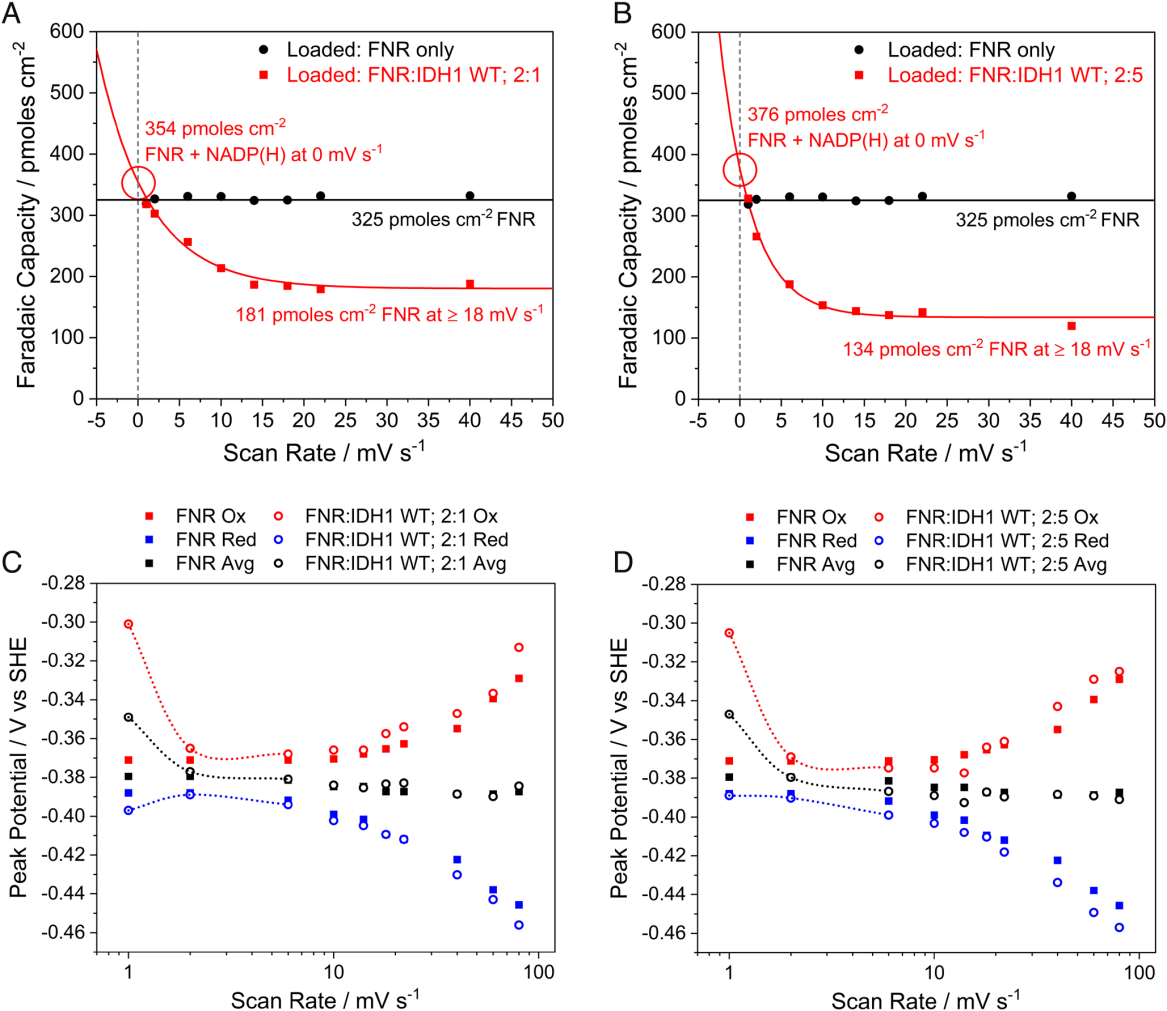
Scan rate-dependent Faradaic capacity (*A* and* B*) and trumpet plots (*C* and* D*) from thin-film voltammetry experiments using electrodes coloaded with FNR and wild-type IDH1 compared to an FNR-only electrode. At low scan rates, the NADP(H) carried in with IDH1 can be detected; peaks collapse to the FNR-only signal at high scan rates. (*A* and *B*) Scan rate-dependent coverage plots fitted with an asymptotic exponential equation to allow extrapolation to 0 mV/s. (*C* and *D*) Trumpet plots showing the changes in oxidation and reduction peak potentials as a function of scan rate (see trend lines). Conditions: stationary (FNR + IDH1)@ITO/PGE electrode (except for FNR-only data, which did not contain IDH1), electrode area 0.06 cm^2^, temperature 25 °C, volume 4 mL, pH = 8 (100 mM HEPES), and enzyme loading ratios (molar): (*A* and* C*): FNR/IDH1; 2/1; (*B* and* D*): FNR/IDH1; 2/5.

Applying the principles of thin-film voltammetry (in which all redox-active species are confined to the electrode surface layer) (43), we determined the amount of NADP(H) trapped in the pores and thus estimated the quantity of IDH1 that carried the NADP(H) in. Aliquots of dimeric wild-type IDH1 (0.85 nmol) were loaded onto two electrodes with different amounts of FNR to give ratios FNR:IDH1 = 2:1 and FNR:IDH1= 2:5 (Fig. 4, panels *A* and *B*, respectively). We then investigated how the apparent electroactive coverage (measured using cyclic voltammetry) (8) varied with the scan rate compared to an electrode loaded only with FNR. Importantly, when loaded alone, the FNR coverage is constant regardless of the scan rate, and the peak currents vary linearly with the scan rate, as expected (*SI Appendix*, Fig. S8). However, when FNR and IDH1 are coloaded, the apparent coverage (now referred to as “Faradaic capacity” to emphasize the fact that the charge passed under a voltammetric peak is time-dependent) increases markedly at very low scan rates (Fig. 4 *A* and *B*). The corresponding trumpet plots (Fig. 4 *C* and *D*) show that at the lowest scan rates, the average of the oxidation and reduction peaks (see trend lines) shifts to a more positive value closer to the NADP^+^/NADPH reduction potential (−0.35 V at pH = 8): in addition, the peak currents are no longer linear with the scan rate (*SI Appendix*, Fig. S8). These observations reveal the presence of a trapped substrate of FNR [in this case NADP(H)] that can be detected provided the scan rate is sufficiently low to allow time for NADP(H) to be transiently released from IDH1 for cycling by FNR. Because NADP(H) is absent from the bulk solution, its loading can be estimated by extrapolating the Faradaic capacity back to 0 mV/s, a hypothetical condition allowing time for all NADP(H) to be released from IDH1 and oxidized/reduced by FNR. Likewise, the actual electroactive FNR coverage is determined for each FNR+IDH1 electrode at high scan rates as the signal reverts to “FNR-only” (Fig. 4 *A* and *B*) (44). Analogous experiments were carried out using IDH1 R132H (*SI Appendix*, Fig. S9). With the knowledge of the amount of copurified NADP(H) in the IDH1 used for electrochemistry (*SI Appendix*, Fig. S2), it was possible to measure the approximate quantities of NADP(H) and IDH1 (or IDH1 R132H) present in the electrode nanopores along with the amount of FNR.

The nanoconfined concentrations of each enzyme were estimated using two further assumptions, i.e., a 3 to 6 µm layer of ITO (42) and a 50% ITO packing volume (45). The results, collated in Table 1, are of interest. First, the nanoconfined enzyme concentrations are very high (millimolar range); indeed, based on the average center-to-center distance in a 1 mM solution being approximately 12 nm (46), the FNR and dimeric IDH1 molecules (39 and 93.4 kDa, respectively) must be highly crowded. The measurements reflect trends in the loading ratios that were used, and the much higher concentration that is found for FNR when loaded alone is not surprising.

Using the data shown in Table 1 as a guide, the total turnover number (TTN) for NADP(H) was estimated for the wild-type IDH1 experiment, as shown in Fig. 3, where a large electrode (4 cm^2^ area) was used instead. Scaling the amount of wild-type IDH1 quantified on the 0.06 cm^2^ electrode from Fig. 4*A* (in which the same FNR:IDH1 = 2:1 molar loading ratio was used) and adjusting for the lower FNR coverage on the larger 4 cm^2^ electrode (40 pmol cm^−2^), while assuming the same FNR:IDH1 ratio, the IDH1_dimer_ coverage on the 4 cm^2^ electrode was estimated to be 41.5 pmol cm^–2^. Based on an average NADP(H) active-site occupancy of 46%, the total amount of NADP(H) used in the reaction was 153 pmol. Taking the average between total charge passed and cumulative yield of 2OG (by NMR) gives a TTN_NADP(H)_ of approximately 160,000.

**Table 1.** Quantities of enzymes and NADP(H) determined in the electrode nanopores and their estimated nanoconfined concentrations

Enzymes present	Loading ratio	FNR/pmol cm^−2^	NADP(H)/pmol cm^−2^	IDH1_dimer_/pmol cm^−2^	[FNR]_pore_/mM	[NADP(H)]_pore_/mM	[IDH1_dimer_]_pore_/mM
FNR	–	325	–	–	1.08–2.17	–	–
FNR, IDH1	2:1	181	173	188	0.60–1.21	0.58–1.15	0.63–1.25
FNR, IDH1	2:5	134	242	263	0.45–0.89	0.81–1.61	0.88–1.75
FNR, R132H	1:2	136	568	291	0.45–0.91	1.89–3.79	0.97–1.94

Note: Concentrations of species in the electrode nanopores ([X]_pore_) were calculated assuming a 3 to 6-µm thick ITO layer and a 50% packing volume (see text). IDH1_dimer_ values were calculated from quantified NADP(H) based on the measured NADP(H):IDH1_dimer_ stoichiometry (46% active site occupancy for wild-type IDH1 and 97.5% occupancy for IDH1 R132H) (see text, *SI Appendix,* Fig. S2). Approximate maximum error limits are as follows: FNR coverage, ±10%; NADPH coverage, ±15%; IDH1 coverage ±20%; and concentrations ±25%.

#### Enzymes and NADP(H) Remain Nanoconfined despite Large Volume Buffer Exchanges.

**Fig. 5.**
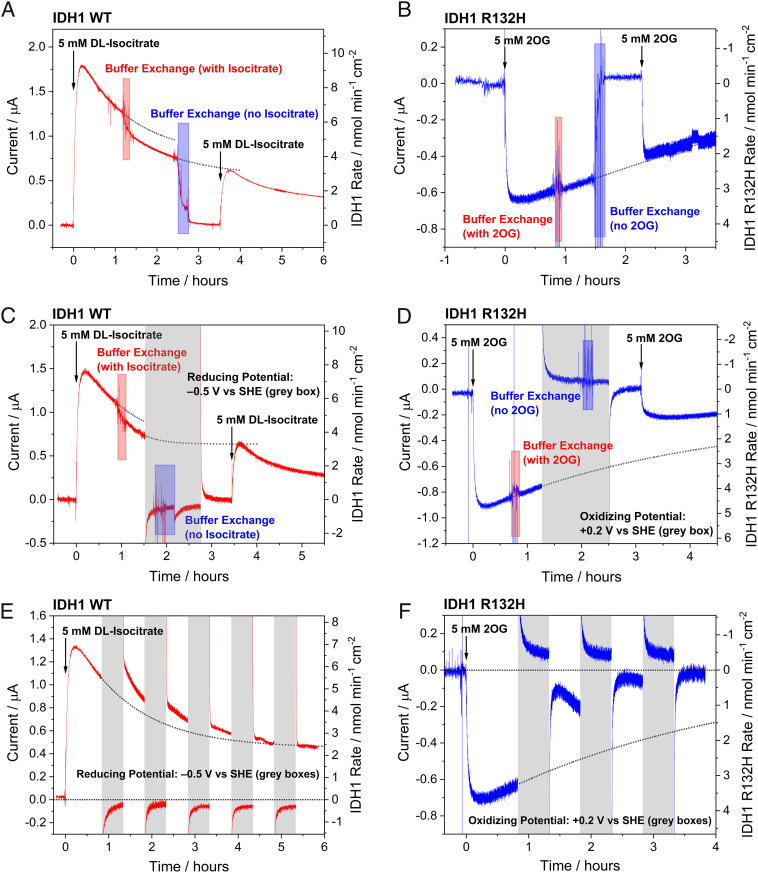
Chronoamperometry experiments showing steady-state catalysis by wild-type IDH1 (isocitrate oxidation to 2OG) and IDH1 R132H (2OG reduction to 2HG) using only copurified enzyme-bound NADP(H), interrupted by live buffer exchanges and potential switches. In panels *A*–*D*, the first buffer exchange (>1,000-fold dilution) used the starting buffer solution with 5 mM substrate (*DL*-isocitrate or 2OG) added to maintain IDH1 catalysis, while the second buffer exchange (~55,000-fold dilution) was performed using the starting buffer solution without any added substrate. (*A*) and (*C*) are equivalent experiments except that the potential in (*C*) was switched from oxidizing (+0.2 V) to reducing (−0.5 V) (timespans indicated by gray boxes) to convert all of the nicotinamide in the pores to NADPH. (*B*) and (*D*) are equivalent experiments with the exception of the potential switch to an oxidizing value (+0.2 V). (*E* and *F*) Oscillating potential switch experiments showing that wild-type IDH1 has a high affinity for both NADP^+^ and NADPH (panel *E*), whereas IDH1 R132H has a much lower affinity for NADP^+^ compared to NADPH (panel *F*). Conditions: (FNR + E2)@ITO/PGE electrode (where E2 represents wild-type IDH1 or IDH1 R132H), area 0.06 cm^2^, rotated at 1,000 rpm, temperature 25 °C, volume 2.6 mL, potential *E* (vs. SHE) = +0.2 V for wild-type IDH1 or –0.5 V for IDH1 R132H (except where potential switches are indicated by gray boxes), and pH = 8 (100 mM HEPES, 10 mM MgCl_2_). Enzyme loading ratios (molar): FNR/IDH1 WT; 2/1 or FNR/IDH1 R132H; 1:2.

To investigate if retention of copurifying NADP(H) in the electrode nanopores depends on whether or not the enzymes are engaged in turnover, buffer exchange experiments were performed using both wild-type IDH1 and IDH1 R132H (Fig. 5 *A* and *B*). After initiating the reaction by introducing isocitrate or 2OG (for wild-type IDH1 and IDH1 R132H, respectively) and achieving a steady-state current, the cell solution was serially exchanged (>1,000-fold dilution) during the experiment with a solution of the same composition [also without NADP(H)]. Following the reaction buffer exchange, the rate of catalysis had only decreased slightly (~15%) for the wild-type (oxidation) and not at all for R132H (reduction) compared to that recorded prior to buffer exchange. These observations imply that the enzymes and NADP(H) responsible for the observed current are confined in the electrode nanopores. A second buffer exchange was then performed to test the hypothesis that the NADP(H) was only trapped in the nanopores due to its rapid shuttling between IDH1 and FNR and that it could escape the pores if this coupling was paused. The buffer solution was serially diluted ~55,000-fold with the initial starting buffer solution (this time without substrate) to remove the substrate (isocitrate for wild-type IDH1 or 2OG for R132H). Following the second buffer exchange, the current dropped to zero, and the electrode was rotated at 1,000 rpm for about 45 min to promote dispersion of any “loosely held” NADP(H) into bulk solution (11). In both cases, when fresh isocitrate (or 2OG) was injected into the cell, the current quickly increased to the value predicted had no intervention been made. The implication of these observations is that both wild-type IDH1 (under oxidizing conditions) and IDH1 R132H (under reducing conditions) can efficiently rebind NADP^+^ or NADPH, respectively, after catalysis is paused (presumably reverting to the open inactive “resting” conformation), otherwise the NADP(H) would escape the pores and no longer be available for catalysis.

#### During a Temporary Pause in Turnover, IDH1 R132H Binds NADPH but Not NADP^+^, Whereas Resting Wild-Type IDH1 Binds Both NADPH and NADP^+^.

Having established that wild-type IDH1 and IDH1 R132H can tightly rebind NADP^+^ and NADPH, respectively, experiments were performed to examine how the oxidation (hydrogenation) state of the pore-confined NADP(H) affects its binding when turnover is paused (Fig. 5 *C*–*F*). To test whether wild-type IDH1 rebinds NADPH tightly, a buffer exchange experiment was performed, in which the potential was reversed from an oxidizing value (+0.2 V) to a reducing value (−0.5 V) prior to the second buffer exchange, thus preventing FNR from recycling NADP^+^ for use by IDH1 and converting all nanoconfined NADP^+^ to NADPH (Fig. 5*C*). After performing the buffer exchange under reducing conditions and rotating the electrode at 1,000 rpm to help disperse any loosely held NADPH as before, the potential was switched back to +0.2 V and the current was allowed to stabilize. Isocitrate was then injected into the solution, initiating a current increase that reached the level predicted had no intervention been made. This observation implies that wild-type IDH1 tightly rebinds NADPH as well as NADP^+^ when the substrate is removed.

The same experiment was then repeated with IDH1 R132H, except that a reducing potential (−0.5 V) was applied to drive 2OG reduction before switching to an oxidizing potential (+0.2 V) prior to the second buffer exchange in order to convert all NADPH in the pores into NADP^+^ (Fig. 5*D*). By contrast with wild-type IDH1, when more 2OG was injected into solution, the current resumed at a level that was < 50% of that expected by extrapolation indicating that IDH1 R132H binds NADP^+^ much less tightly than NADPH.

To confirm whether the oxidation state of NADP(H) determines its affinity for resting state IDH1 R132H, whereas this condition is not so critical for wild-type IDH1, we carried out oscillating potential switch experiments for both enzymes. In each case, a steady-state current was established before the potential was switched between oxidizing and reducing values in hour-long cycles (30 min at each potential) (Fig. 5 *E* and *F*). For wild-type IDH1, each time the potential was switched from +0.2 V to −0.5 V to convert all the copurifying NADP^+^ into NADPH and ablate catalytic action, normal oxidation resumed when the potential was switched back to +0.2 V. The opposite was true for IDH1 R132H, where application of an oxidizing potential to convert all the NADPH into NADP^+^ had a detrimental effect on activity. The current decreased by over 50% after the first cycle before losing all activity after just three cycles, confirming that IDH1 R132H binds NADPH much more tightly than NADP^+^. In revealing such on/off redox-state dependence, the e-Leaf thus operates like an “electromagnetic gripper,” releasing its cargo as the voltage is changed.

These results show that wild-type IDH1 can bind both NADP^+^ and NADPH tightly when the enzyme is in its inactive state (Fig. 5 *A*, *C*, and *E*). This state thus has an extremely high affinity for NADP(H) that is not represented by the reported *K*_m_ values of 27 µM for NADP^+^ and 115 µM for NADPH measured under steady-state conditions for oxidation of isocitrate and reduction of 2OG, respectively (20). By contrast, although NADPH is bound sufficiently tightly by IDH1 R132H to remain trapped within the nanopores (Fig. 5*B*), NADP^+^ is able to escape (Fig. 5 *D* and *F*). These results are consistent with the NMR data, Fig. 1 *A* and *B*, showing that wild-type IDH1 copurifies (in the “resting” open inactive conformation) with a mixture of NADP^+^ and NADPH bound, whereas IDH1 R132H copurifies exclusively with NADPH. Such selectivity in cofactor retention also reflect the reactions that each enzyme performs: wild-type IDH1 catalyzes the reversible NADP(H)-dependent isocitrate to 2OG reaction, while IDH1 R132H catalyzes the irreversible NADPH-dependent reduction of 2OG to 2HG, the 2OG/2HG reduction potential being much more positive than the value for the NADP^+^/NADPH couple (8). The fact that the R132H variant can easily replace NADP^+^ by NADPH whenever turnover is paused may have biological relevance as it may assist the enzyme in performing the cancer-associated 2OG reduction reaction.

#### Recycling under Nanoconfinement Greatly Increases the Efficiency with Which NADP(H) Is Used in Enzyme Cascade Catalysis.

**Fig. 6.**
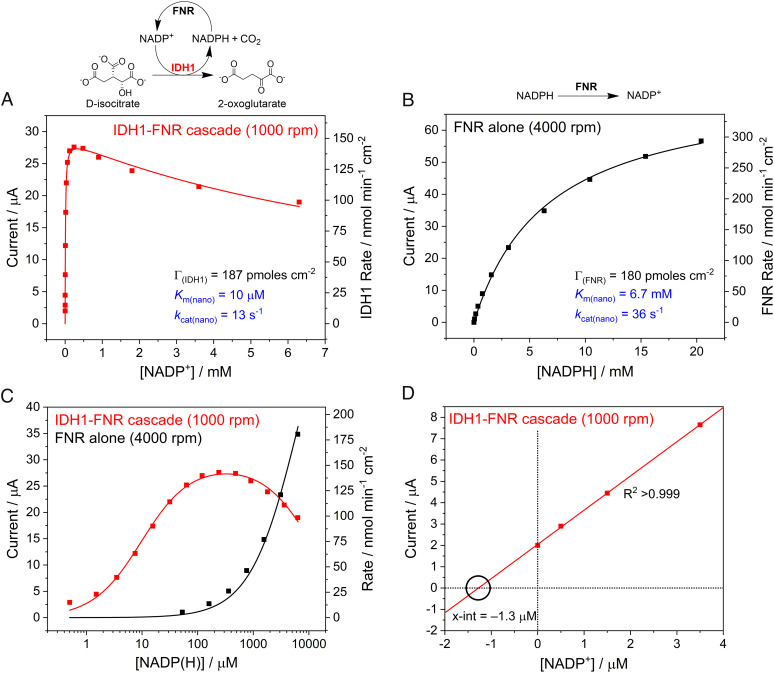
Nanoconfined enzyme kinetics measuring the activity of the IDH1-FNR cascade (*A*) and FNR alone (*B*) fitted using derived electrochemical-kinetic equations [Eq. **2** (*A*) and Eq. **1** (*B*)]. The same electrode (with FNR and IDH1 loaded in a 2:1 ratio) was used in (*A*) and (*B*): (*A*) Rate of isocitrate oxidation at increasing concentrations of NADP^+^, where NADP(H) is recycled between IDH1 and FNR. (*B*) Rate of NADPH oxidation by nanoconfined FNR at increasing concentrations of NADPH. (*A* and *B*) The enzyme coverages (shown in black) were used as inputs for the fitted equations: results (shown in blue) were determined by fitting the equations to the data. The data in (*A*) were fitted using Eq. **2** to account for the slight substrate inhibition observed (*K*_i(nano)_ = 11 mM). Both equations fitted to the data sets had an R^2^ > 0.99. (*C*) Data from (*A*) and (*B*) compared on a semilog plot. (*D*) The first four data points in panel *A* were extrapolated to the x-intercept to estimate the effective NADP(H) solution concentration of the nanoconfined IDH1-copurified NADP(H) (see text). Conditions (*A* and *B*): (FNR + IDH1)@ITO/PGE electrode, area 0.06 cm^2^, temperature 25 °C, volume 4 mL, potential *E* (vs. SHE) = +0.2 V, pH = 8 (100 mM HEPES), and enzyme loading ratios (molar): FNR/IDH1; 2/1. (*A*): buffer also contained: 10 mM MgCl_2_, 10 mM DL-isocitrate; rotated at 1,000 rpm. (*B*): rotated at 4,000 rpm.

An important advantage of nanoconfinement for cascades is the potential for temporal retention of intermediates and exchangeable cofactors (their rates of escape are lower than the rates at which they are processed or recycled). The FNR/IDH1/NADP(H) system presented a special opportunity to quantify this advantage by comparing the catalytic action of FNR at various external NADPH levels in the presence or absence of coupling with IDH1. The energetics of the catalytic oxidation of NADPH by FNR, and of the coupling to isocitrate oxidation via IDH1, have been presented previously (8, 12). Two experiments were carried out using the same electrode, coloaded with FNR and wild-type IDH1, in order to minimize differences due to different ITO thicknesses and enzyme loadings (Fig. 6). In the first experiment, the current was due to catalytic isocitrate oxidation mediated by NADP(H) recycling between IDH1 and FNR: as NADP^+^ was introduced, the current increased steeply to a maximum value before decreasing gradually (Fig. 6, panel *A*). After completing the isocitrate oxidation experiment, the cell solution was exchanged, the electrode was rinsed to remove any residual isocitrate, Mg^2+^, and NADP^+^, and the experiment was repeated. This time, however, NADPH was titrated into the cell solution without isocitrate or Mg^2+^ present; therefore, the current was due to the catalytic oxidation of NADPH by FNR without coupling to IDH1 (Fig. 6, panel *B*). For the latter experiment, the data were fitted to a modified form of the Michaelis–Menten equation (*SI Appendix*),

[1] $$I=\frac{nAF\Gamma k_{cat(nano)}\left[S\right]}{K_{m(nano)}+\left[S\right]},$$

where *I* is the measured current (Amps), *n* is the number of electrons transferred per molecule, *A* is the electrode surface area (cm^2^), *F* is the Faraday constant (96,485 C per mole of electrons), [*S*] is the NADP(H) concentration, *Γ* and *k*_cat(nano)_ are the enzyme coverage (mol cm^−2^) and maximum turnover frequency (s^−1^) of the rate-determining enzyme, and *K*_m(nano)_ is the apparent Michaelis constant (mM) for NADP(H) under nanoconfinement. For panel *A*, a term was included to take into account the inhibition (*K*_i_) at high [NADP^+^] (Eq. **2** and *SI Appendix*).

[2] $$I=\frac{nAF\Gamma k_{cat(nano)}\left[S\right]}{K_{m(nano)}+\left[S\right]+\frac{{\left[S\right]}^{2}}{K_{i}}}.$$

Importantly, *K*_m(nano)_, which reflects how efficiently NADP^+^ or NADPH is used by the nanoconfined system, is 650-fold higher (6.5 mM vs. 10 μM) when IDH1 is catalytically redundant (since isocitrate is not present), i.e., where NADPH is being used as the sole reactant rather than as a rapidly recycling cofactor. The current due to the FNR-only reaction (Fig. 6*B*) was sensitive to the electrode rotation rate even at 4,000 rpm (the rate thus being limited by the supply of NADPH from bulk solution) whereas that for the IDH1-FNR cascade reaction (Fig. 6*A*) did not increase above 1,000 rpm, consistent with internal NADP^+^/NADPH recycling being rate limiting. The massive gain in the efficiency of cofactor usage under nanoconfined recycling is clear (Fig. 6*C*), with an enhancement of more than two-orders of magnitude based on the NADP(H) concentration required, while the maximum rate (current) measured for the FNR-only reaction is just two to threefold higher than the IDH1-FNR cascade reaction. On the basis of the estimated enzyme coverages (Table 1), the *k*_cat(nano)_ values were estimated to be 13 s^−1^ for the data shown in Fig. 6*A* and 36 s^−1^ for the data shown in Fig. 6*B*, corresponding to the maximum turnover frequencies of IDH1 and FNR, respectively, under nanoconfinement. For comparison, solution assays performed under comparable conditions gave *k*_cat_ values of 86 s^−1^ for IDH1 (41) and 51 s^−1^ for FNR (*SI Appendix*, Fig. S10).

An alternative way of expressing the efficiency gain due to cofactor nanoconfinement is to determine the bulk solution concentration that would be required to produce the same activity as the NADP(H) taken into the electrode nanopores. The IDH1-copurified NADP(H) probably contributes slightly to the lower *K*_m(nano)_ (Fig. 6*A*) because the first of the data points was measured after isocitrate was injected into the solution before any NADP^+^ was added (only the copurified NADP(H) was present, giving 2 µA current). Fig. 6*D* shows a linear extrapolation of the first four data points in Fig. 6*A* to the x-intercept (since *I* = 0 when [NADP(H)] = 0 and *I* α [*S*] when [*S*] ≪ *K*_m(nano)_). The intercept indicates that the copurified NADP(H) trapped in the nanopores has an effective bulk solution concentration of 1.3 µM [the solution concentration required to achieve the same current as copurified NADP(H)]. Comparison of this value with the estimated NADP(H) pore concentration of 0.58 to 1.15 mM (Table 1) reveals that the nanoconfined IDH1/FNR system concentrates NADP(H) by 450 to 900-fold compared to the bulk solution, greatly increasing the efficiency of its use.

It was instructive to follow up these experiments by designing a reasonable “like-for-like” comparison (*SI Appendix*, Fig. S11) between the IDH1-FNR cascade nanoconfined within the electrode nanopores and the equivalent quantity of enzyme dispersed in dilute solution without any added NADP(H). The solution experiments involved taking the same quantities of enzyme loaded under electrode nanoconfinement for the analytical-scale or larger-scale measurements described above, diluting into 4 mL of solution, and measuring the quantity of 2OG formed after 12 h by driving the reaction entirely homogeneously using a large excess of benzyl viologen (to oxidize FNR in place of the electrode, providing NADP^+^ recycling). Importantly, the comparison was based on the amount of product made by a given amount of enzyme normalized to the reaction volume (electrochemical experiments were also carried out in 4 mL bulk solution). The large electrode used for Fig. 3, containing a solution equivalent of 40 nM FNR and 41.5 nM IDH1_dimer_, produced 8-fold more 2OG than the solution reaction. Even the analytical-scale electrode, which contained the solution equivalent of only 2.6 nM FNR and 2.7 nM IDH1_dimer_, produced 50 µM 2OG, while no product was detected in the equivalent solution assay. Thus, for a given minuscule quantity of NADP(H), the nanoconfined system offers a clear kinetic advantage over one that is dispersed despite the significant disadvantage of decreased mass transport efficiency due to its heterogeneous nature.

## Conclusions

The combined results show that nanoconfinement of the IDH1-FNR/NADP^+^-NADPH cascade enables a massive increase in the efficiency with which NADP(H) is used as a recycling cofactor. Within the nanopores, NADP(H) is retained and concentrated with respect to the external environment; by contrast, small substrates and “end” products [e.g., isocitrate, 2OG and 2HG (for R132H)] must have relatively free passage in and out of the pores (otherwise no catalytic current would be observed). Together with recent results involving ATP recycling (47) and a four-enzyme linear cascade in which intermediates were largely retained in the electrode pores (9), our conclusion that efficient cofactor recycling is a crucial aspect of, at least some, efficient catalytic cascades may have wider significance. Exchangeable cofactors like NAD(P)(H), ATP/ADP/AMP, and others are often treated as (co)substrates for single enzymes, but this view is likely misleading, as within the context of metabolism they are intermediates that are likely recycled, within a (at least partially) confined catalytic cascade system. Dispersed, diluted cofactors may be largely redundant and represent a source of inefficiency during catalysis.

The results have potentially compelling relevance to conditions existing in subcellular compartments, where enzyme concentrations may be similar to (or may exceed) that of their substrates and intermediates, and a steady-state is attained much more rapidly than an equivalent cascade reaction in dilute solution, so helping to avoiding long lag periods (delays in response to stimuli). Even with an error tolerance of 25% (Table 1), the high local enzyme concentrations estimated for our work (0.60 to 1.21 mM FNR and 0.63 to 1.25 mM IDH1_dimer_) approach the physical limits based on available space and are likely to resemble conditions found in some zones of cells as opposed to standard solution assays that use dilute (often nanomolar) concentrations. The very high local enzyme concentrations should greatly increase the overall rates, but only up to a limit as the enzymes now compete for space—this is inferred by the lower FNR coverage when coloaded alongside IDH1 (crowding may also promote inhibitory interactions between enzymes).

The nanoporous metal oxide environment might be far removed from a lipid vesicle or protein-shelled microcompartment, but the advantages of confinement for multistep processes—locally concentrating the sequential catalysts and curtailing premature escape of intermediates—are analogous. The condition may also be relevant to precellular evolution, as the formation of organic molecules in stepwise reactions of stable catalytic cycles could have exploited cavities in minerals (48, 49).

From a technical standpoint, the exquisite control over enzyme cascades made possible with the e-Leaf methodology can be exploited to gain interesting insight into enzyme function and mechanism. The precise control of the NADP(H) status that can be achieved virtually instantaneously by switching or cycling the electrode potential made it possible to establish, definitively, that the substrate-free resting state of wild-type IDH1 binds both NADP^+^ and NADPH very tightly, whereas R132H selects for NADPH. These results would have been difficult to obtain using other methods and are of interest in developing a general understanding of why cancer-associated IDH1 and IDH2 variants preferentially catalyze the neomorphic 2OG reduction reaction over the wild-type isocitrate oxidation reaction in cells. Beyond IDH1, the affinities and rate constants for the binding of (co)substrates/(co)products to different catalytically active and inactive forms of enzymes are clearly important: in addition to the obvious relevance for substrate/product inhibition, extremely high affinities of particular enzyme forms for exchangeable cofactors (manifested as copurification with NADP(H) in the case of IDH1) may reflect a property evolved to optimize use of local concentrations within cells. Currently, obtaining such detailed biochemical information is technically difficult due to the transient nature of many enzyme states. New methods, such as that described here, may help reveal these important features.

On a final note, bioinspired catalysis has long been focused on mimicking—structurally, functionally, or both—the chemistry associated with individual enzyme active sites. The results presented here highlight the potential for mimicking a higher level of metabolic organization—involving the identification and exploitation of protein and non-protein catalysts along with electrocatalysts for challenging multistep processes.

## Materials and Methods

Ferredoxin NADP^+^-reductase (FNR) from *Chlamydomonas reinhardtii* and human IDH1 (wild-type and R132H) were expressed and purified as previously described (8, 41). Nanoporous ITO electrodes were prepared as described previously (8), and the enzymes were loaded by applying concentrated mixtures (at the specified ratios) to the electrode surface for approximately 45 min before rinsing thoroughly with buffer solution. Electrochemical experiments were performed in an anaerobic glove box (Braun Technologies) using an Autolab PGSTAT 10 potentiostat and Nova software. In-house custom glass cells were used for all experiments. All routine procedures and conditions along with special procedures for solution kinetic assays, ^1^H NMR spectroscopy, and non-denaturing mass spectrometry are described in detail in the *SI Appendix*. Derivations of equations are also given in the *SI Appendix*.

## Supplementary Material

Appendix 01 (PDF)

## Data Availability

All study data are included in the article and/or *SI Appendix*.
