# Supplementary material for: NADP(H)-dependent biocatalysis without adding NADP(H)
Source: Proc Natl Acad Sci U S A. 2022 Dec 27;120(1):e2214123120. doi: 10.1073/pnas.2214123120 (PMC9910440; doi:10.1073/pnas.2214123120)
Supplement: Supplementary file 1 — Appendix 01 (PDF) [file pnas.2214123120.sapp.pdf]

Supporting Information

NADP(H)-dependent biocatalysis without adding NADP(H)

Ryan A. Herold[1], Raphael Reinbold[2], Christopher J. Schofield[2], and Fraser A. Armstrong[1]

[1]Department of Chemistry, University of Oxford, Oxford OX1 3QR, United Kingdom.

[2]Department of Chemistry and the Ineos Oxford Institute for Antimicrobial Research, University of Oxford, Oxford OX1 3QY, United Kingdom.

Materials and Methods

Enzyme Expression and Purification

Ferredoxin $NADP^+$-reductase (FNR) from *Chlamydomonas reinhardtii* and human isocitrate dehydrogenase 1 (wild-type and R132H) were expressed and purified as previously described (1, 2).

Electrode Fabrication

Nanoporous indium tin oxide (ITO) electrodes were made by electrophoretic deposition of ITO nanoparticles (<50 nm, Sigma-Aldrich) onto either pyrolytic graphite edge (PGE) or titanium foil electrodes as previously described (1), except that the deposition time was increased to 8–12 minutes from 6 minutes in order to produce a thicker ITO layer.

Enzyme Loading

Enzymes were loaded onto nanoporous ITO electrodes as previously described (1). Briefly, a concentrated mixed enzyme solution (4 μL for 0.03 or 0.06 cm^2 PGE electrodes; 18 μL for 4 cm^2 Ti foil electrode) was incubated on the electrode for ~45 minutes at room temperature before being rinsed thoroughly to ensure that any unbound enzyme was removed. For PGE electrodes, 0.85 nmoles of IDH1 (both wild-type and R132H) was used, and the amount of FNR was adjusted to achieve the desired molar loading ratio. Four-fold more enzyme (3.4 nmoles wild-type IDH1 and 6.8 nmoles FNR) was used to load the scaled-up 4 cm^2 Ti foil electrode described in **Figure 3**. Enzyme molar ratios were calculated based on IDH1 homodimers.

Electrochemical Quantification of Adsorbed FNR

Electroactive FNR was quantified using cyclic voltammetry as previously described (1).

Electrochemical Experiments

Electrochemical experiments were performed in an anaerobic glove box (Braun Technologies) with a nitrogen atmosphere (O_2 < 1 ppm) using an Autolab PGSTAT 10 potentiostat and Nova software. In-house custom glass cells were used for all experiments; a two-chamber glass cell was used for experiments with an ITO/PGE rotating disc electrode (the working electrode and platinum counter electrode were in the same chamber) (1, 3), while a

three-chamber glass cell was used for the scaled-up experiment with the working, counter, and reference electrodes housed in separate chambers (4). In the scaled-up experiment (three-chamber cell), the counter electrode and working electrode chambers were separated by a glass frit and contained the same buffer solution. In all experiments, the reference electrode chamber contained 0.1 M NaCl and was connected to the working electrode chamber via a Luggin capillary. Electrode potentials were measured against a saturated calomel electrode (SCE) and converted to SHE using a SCE to SHE conversion table (1).

Live Buffer Exchange Protocol

Live buffer exchanges (i.e., where current is continuously monitored during the buffer exchange) were performed as previously described (1), except that different volumes of exchange buffer were used depending on how much dilution was required. Briefly, a 1 mL syringe was used to reduce the reaction volume to ~1.5 mL while making sure that the electrode was always submerged in order to maintain electrical contact. A 60 mL syringe containing fresh buffer solution (30 mL buffer for >1000-fold dilution or 47 mL for ~55000-fold dilution) was then used to slowly inject 8 mL of buffer into the cell. The syringe was removed, and a 20 mL syringe was then used to remove 8 mL of buffer from the cell now containing the diluted starting buffer. Once the buffer volume was reduced to ~1.5 mL again, 8 mL of buffer solution was again added to the cell, and the process was repeated until all of the exchange buffer was used and the final cell volume was equal to the starting volume. The electrode rotation (1000 rpm) provided adequate solution agitation to ensure mixing during the procedure.

IDH1-FNR Experiments in Dilute Solution

The isocitrate oxidation activity of the IDH1-FNR cascade was measured in dilute solution both with added NADPH (positive control) and without (only IDH1-copurified NADP(H) present) over 12 hours for comparison with the same cascade reaction nanoconfined in electrodes. Two experiments were carried out at different enzyme concentrations based on the amount of FNR and $IDH1_{dimer}$ that was quantified on individual small (0.06 cm^2) and large (4 cm^2) electrodes used in experiments (see **Supporting Figure 11** for details), equivalent to 2.6 (2.7) and 40 (41.5) nM of FNR (parentheses denote [$IDH1_{dimer}$]) in a 4 mL solution. The solution reactions were allowed to run at 25 °C for 12 h before heat quenching and analysis by ^{1}H NMR. $NADP^+$ was recycled by FNR for use by IDH1, with a large excess of benzyl viologen in solution (25 mM) to continuously re-oxidize FNR in place of the electrode. The cascade activity in solution was determined based on the amount of product made (2OG) in the solution over a 12-hour period (quantified by ^{1}H NMR after heat quenching). The molar ratio of FNR:$IDH1_{dimer}$ used was 1:1.04 ([IDH1] = 1.04 x [FNR]), the same ratio that was quantified in the electrode nanopores for the relevant electrochemical experiments using the FNR:$IDH1_{dimer}$ = 2:1 loading ratio (**Table 1**). Other conditions: 400 µL reaction volume, 100 mM HEPES (pH 8), 10 mM $MgCl_2$, 5 mM *D*-isocitrate, 25 mM benzyl viologen, 25 °C. All reactions were performed in triplicate with error bars representing the standard deviation.

FNR Solution Assays

The NADPH oxidation activity of FNR in dilute solution was determined in an anaerobic glovebox (Belle Technologies) (O_2 < 1 ppm) by measuring the absorbance of reduced benzyl viologen (Sigma-Aldrich) at 580 nm (isosbestic point of monomeric and dimeric reduced benzyl viologen) (5) using an Ocean Optics S2000 Fiber Optic spectrophotometer with OOIBase32 software (Ocean Optics, Inc.). An extinction coefficient of 7800 M^{-1} cm^{-1} was used for 1e^- reduced (radical) benzyl viologen (6) (two molecules of benzyl viologen are reduced for every molecule of NADPH oxidized). Reaction conditions: 2 nM FNR, 100 mM HEPES (pH = 8), 5 mM benzyl viologen, 26 °C, [NADPH] was varied, 1 mL reaction volume.

^{1}H Nuclear Magnetic Resonance (NMR) experiments

NMR spectra were obtained using a Bruker AVIII 700 MHz NMR spectrometer equipped with an inverse 5mm TCI ^{1}H/^{13}C/^{15}N cryoprobe. The water signal was suppressed by excitation sculpting. For copurification experiments, proteins were buffer exchanged into NMR buffer (50 mM Tris-d_{11}, 100 mM NaCl, 10 % D_2O, pH 7.5) using Micro Bio-Spin 6 columns (Bio-Rad) according to the manufacturer's protocol. The final protein concentrations were 50 µM for FNR and IDH1 R132H and 124 µM for wild-type IDH1 (a higher concentration was required to compensate for the weaker NADP(H) signal intensity due to copurification with both $NADP^+$ and NADPH). A ^{1}H spectrum (NS: 64, relaxation delay: 2 s) of this solution was obtained before the protein was denatured by heat (2 min, 100 °C). A spectrum of the denatured protein sample was recorded and the copurifying molecules identified by spiking with 100 µM of NADPH, $NADP^+$, or FAD.

A control experiment to investigate oxidation of NADPH to $NADP^+$ in the presence of FAD was set up according to the same parameters. The sample was composed of FAD (1 mM) and NADPH (1 mM) in NMR buffer. An equivalent experiment was set up with benzyl viologen (1 mM) and NADPH (1 mM).

For quantitative NMR experiments, 80 µL of a sample from electrochemical experiments in 100 mM HEPES was mixed with 16 µL D_2O (final 10% concentration), 16 µL 3-(Trimethylsilyl)propionic-2,2,3,3-d_4 acid sodium salt solution (TSP; final: 1 mM), 48 µL MQ water. The concentration of *D*-isocitrate and 2OG was calculated using the concentration of the internal standard TSP. The number of scans was 32, and D1 was 30 s. If the sample concentration was small, the number of scans was increased to 128. The water signal was suppressed by excitation sculpting.

Non-denaturing Mass Spectrometry Experiments

Non-denaturing mass spectrometry studies were conducted as previously reported (2). In brief, the protein samples were exchanged into buffer (ammonium acetate, 200 mM, pH 7.5) with Micro Bio-Spin 6 Columns (Bio-Rad). The samples were analysed at a concentration of 20 µM on a quadrupole-TOF (Waters Synapt G2Si) instrument and an Advion Triversa Nanomate chip-based ESI autosampler. The parameters were as follows: 1.7-1.8 kV, spray backing gas pressure 0.6 psi, inlet pressure 3.7 mbar; cone voltages 100V and 5.2 V.

Supporting Equations

Derivation of Equations 1 and 2 (main text)

In order to derive an equation giving the current for an electroactive enzyme as a function of substrate concentration, we start with the equation describing the current from an electroactive enzyme,

$$I = nAF\Gamma k_{\mathrm{T}}, \qquad \text{Supporting Eq. 1}$$

where I is the current (Amps), n is the number of electrons transferred per molecule, A is the electrode surface area (cm^2), F is the Faraday constant (96,485 C per mole of electrons), Γ is the enzyme coverage (moles cm^{-2}), and k_{T} is the enzyme turnover frequency (s^{-1}). Because the enzyme turnover frequency, k_{T}, changes with substrate concentration as described by the

Michaelis-Menten equation (this equation also can be fitted to currents measured for nanoconfined enzymes in the e-Leaf), the substrate-dependent Michaelis-Menten turnover frequency must be substituted for k_{T}. When written in the form,

$$v = \frac{k_{\mathrm{cat}}[S]}{K_{\mathrm{m}} + [S]}[E], \qquad \text{Supporting Eq. 2}$$

the Michaelis-Menten turnover frequency is

$$k_{\mathrm{T}} = \frac{k_{\mathrm{cat}}[S]}{K_{\mathrm{m}} + [S]} \qquad \text{Supporting Eq. 3}$$

Combining Supporting Eqs. 1 and 3 gives Supporting Eq. 4 (Eq. 1 in the main text), where k_{cat} and K_{m} have been renamed as $k_{\mathrm{cat(nano)}}$ and $K_{\mathrm{m(nano)}}$ to indicate that they are values for nanoconfined enzymes, not values for enzymes in dilute solution.

$$I = \frac{nAF\Gamma k_{\mathrm{cat(nano)}}[S]}{K_{\mathrm{m(nano)}} + [S]} \qquad \text{Supporting Eq. 4}$$

(where $\frac{k_{\mathrm{cat(nano)}}[S]}{K_{\mathrm{m(nano)}}+[S]} = k_{\mathrm{T}}$)

Supporting Eq. 5 is a modified form of Supporting Eq. 4 that was derived to account for substrate inhibition. It was derived the same way as Supporting Eq. 4, except that k_{T} in Supporting Eq. 1 was replaced by $\frac{k_{\mathrm{cat}}[S]}{K_{\mathrm{m}}+[S]+\frac{[S]^2}{K_i}}$, the Michaelis-Menten turnover frequency for a substrate inhibited enzyme, where K_i is the substrate inhibition constant.

$$I = \frac{nAF\Gamma k_{\mathrm{cat(nano)}}[S]}{K_{\mathrm{m(nano)}} + [S] + \frac{[S]^2}{K_i}} \qquad \text{Supporting Eq. 5}$$

Supporting Results

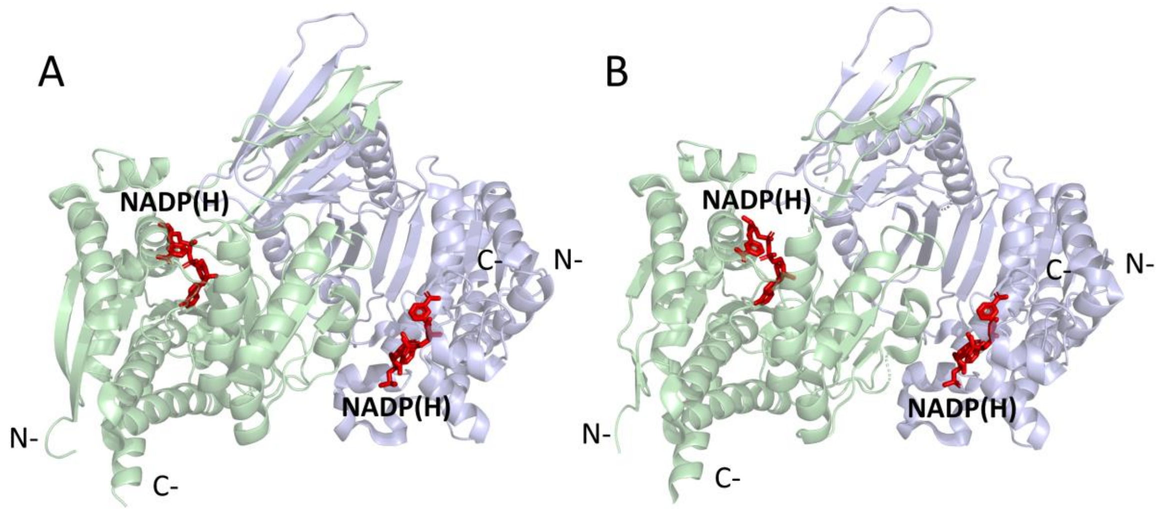

Supporting Figure 1. Crystal structure views of dimeric IDH1 showing copurified NADP(H) bound at each monomer active site. (A) Wild-type IDH1 (PDB: 1T09) (7). (B) Cancer-associated IDH1 R132H variant (PDB: 3MAR) (8). Both enzymes were crystallized without added NADP(H) (7, 8) and show IDH1 in the "open inactive" conformation (9).

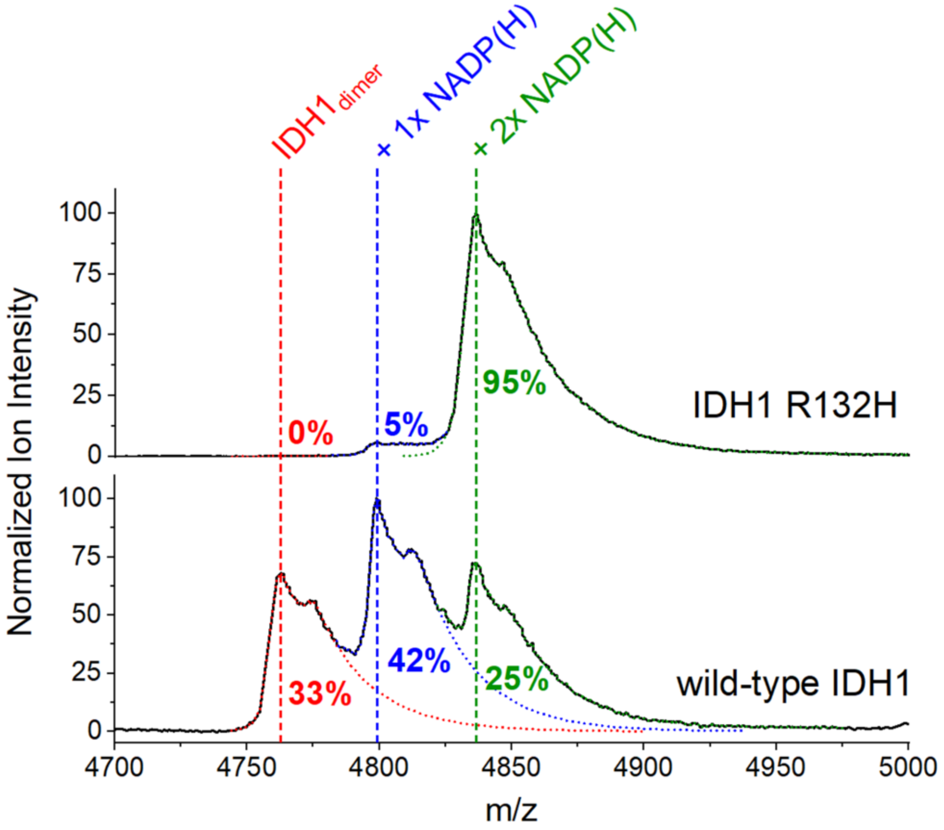

Supporting Figure 2. Non-denaturing mass spectrometry for IDH1 R132H (top) and wild-type IDH1 (bottom) showing the relative fractions of dimeric enzyme with 2 molecules of bound NADP(H) (green), one molecule of bound NADP(H) (blue), and no bound NADP(H) (red) after purification. To separate the overlapping peaks in the bottom panel (wild-type IDH1) and allow integration, the exponential parts of each peak was fit using an asymptotic exponential equation (approaching 0). The decay rate constant used to fit the first two peaks (red and blue) was determined by fitting the same exponential equation to the green peak (2 molecules of bound NADP(H)), the trailing tail of which does not overlap with other peaks.

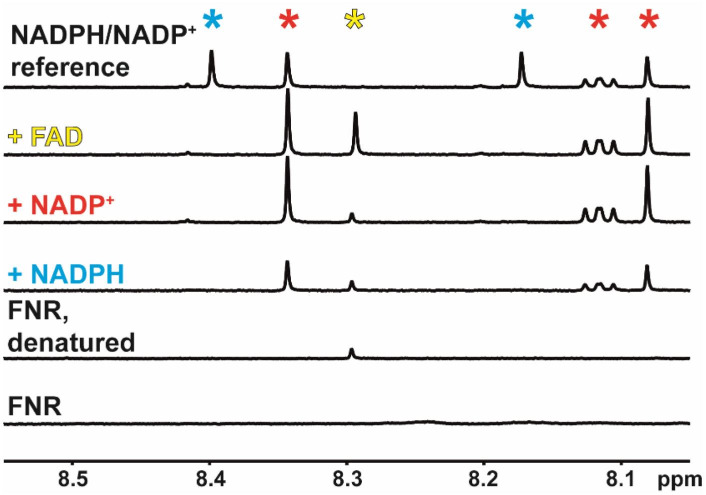

Supporting Figure 3. ^{1}H NMR data showing that no NADP(H) is copurified with FNR. The FAD cofactor only becomes visible after the enzyme is denatured, which was confirmed by adding additional FAD (100 µM) to the NMR tube containing the denatured FNR. Interestingly, the NADPH that was added to the sample (100 µM) was oxidized to NADP$^+$ despite the FNR being denatured. A control experiment (**Supporting Figure 4**) confirmed that "free" FAD in solution is able to directly oxidize NADPH.

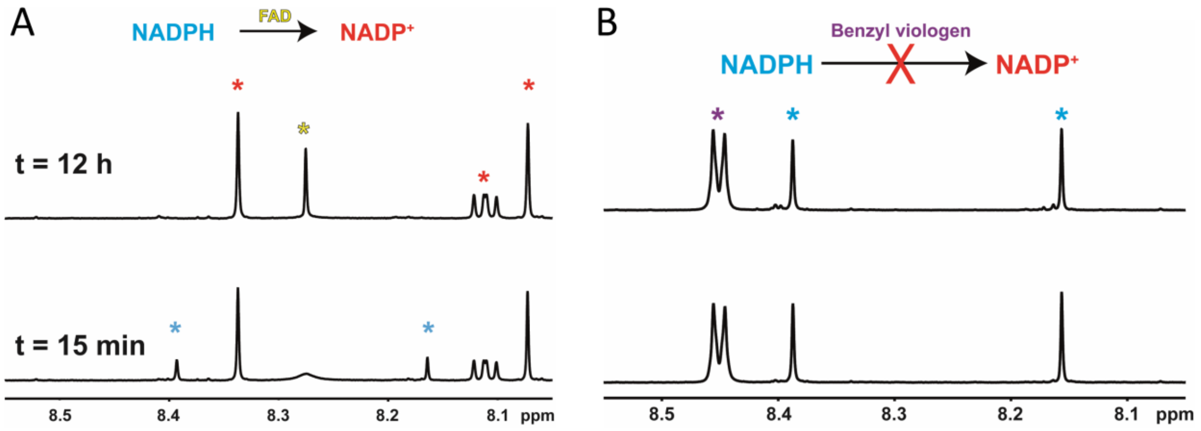

Supporting Figure 4. ^{1}H NMR control experiments to test if either FAD (A) or benzyl viologen (B) were capable of directly oxidizing NADPH in solution. (A) "Free" FAD (100 µM) in solution is able to directly oxidize NADPH (100 µM) in solution. At the t = 15 minutes timepoint (bottom), it was observed that the fully-oxidized FAD peak (denoted with a yellow star) was much smaller and broader than expected, likely due to it having been reduced by NADPH. After 12 hours, the oxidized FAD peak can be clearly seen, having been reoxidized by oxygen, alongside NADP$^+$ (no NADPH remains). (B) Benzyl viologen is not able to oxide NADPH in solution. The bottom scan is after 15 minutes of incubation; the top scan is after 12 hours.

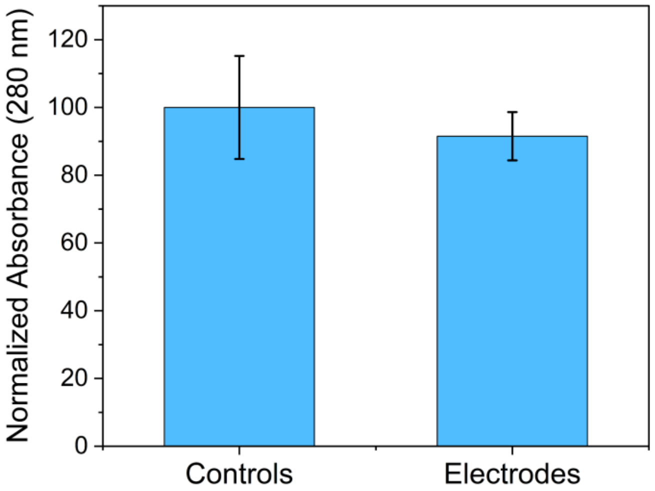

Supporting Figure 5. Bar chart showing no significant difference ($p = 0.45$; two-tailed t-test assuming unequal variances) in the amount of enzyme rinsed from 0.06 cm^2 electrodes (3) after enzyme loading vs control samples (3), where no electrode was used (directly diluted enzyme stock). The three electrodes were loaded with enzyme (1.27 nmoles total; FNR:IDH1 $R132H_{dimer}$; 1:2) following the typical procedure (see **Materials and Methods**). The electrodes were rinsed using buffer (100 mM HEPES (pH = 8)), and the rinsed solution was kept for comparison with three aliquots of the same amount of enzyme that was directly diluted into the same amount of buffer used for rinsing. Based on absorbance at 280 nm, the rinsed solution contains roughly the same amount of enzyme as the directly diluted stocks, confirming that only a tiny fraction of the enzyme used is actually taken up into the electrode nanopores with the vast majority being rinsed off. Each sample was analyzed using three technical replicates. Error bars represent the standard deviation.

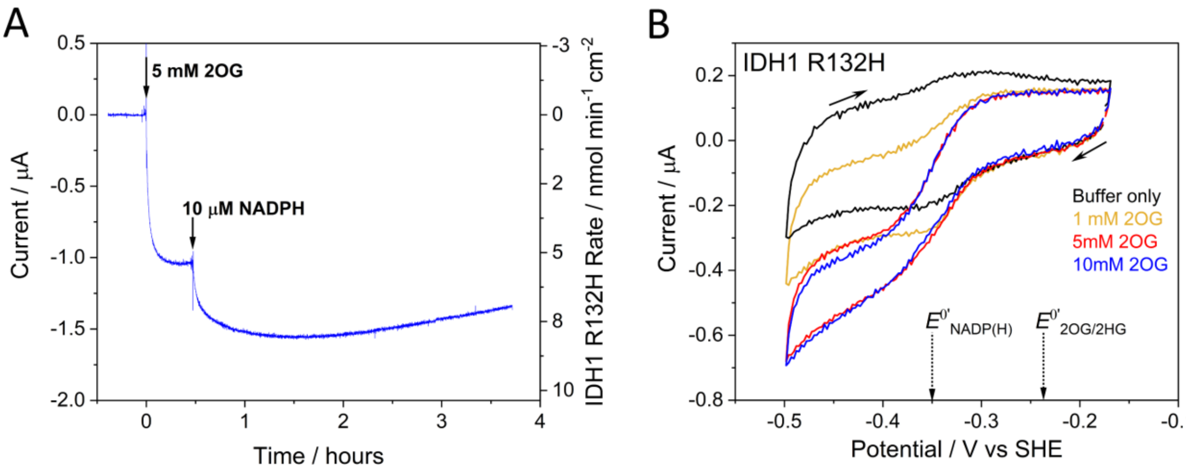

Supporting Figure 6. Electrochemical experiments showing that IDH1 R132H activity is observed using only copurified bound NADP(H) (no NADP(H) was added). (A) Chronoamperogram showing IDH1 R132H activity after 5 mM 2OG was injected into solution followed by an addition of 10 μM NADPH. (B) Cyclic Voltammetry showing IDH1 R132H activity at different concentrations of 2OG. Conditions (A and B): (FNR+IDH1 R132H)@ITO/PGE electrode, area 0.06 cm^2, temperature 25 °C, volume 4 mL, pH = 8 (100 mM HEPES, 10 mM $MgCl_2$), enzyme loading ratios (molar): FNR/IDH1 R132H; 2/5. (A): 1000 rpm, potential E (vs SHE) = –0.5 V. (B): scan rate 1 mV/s, stationary electrode.

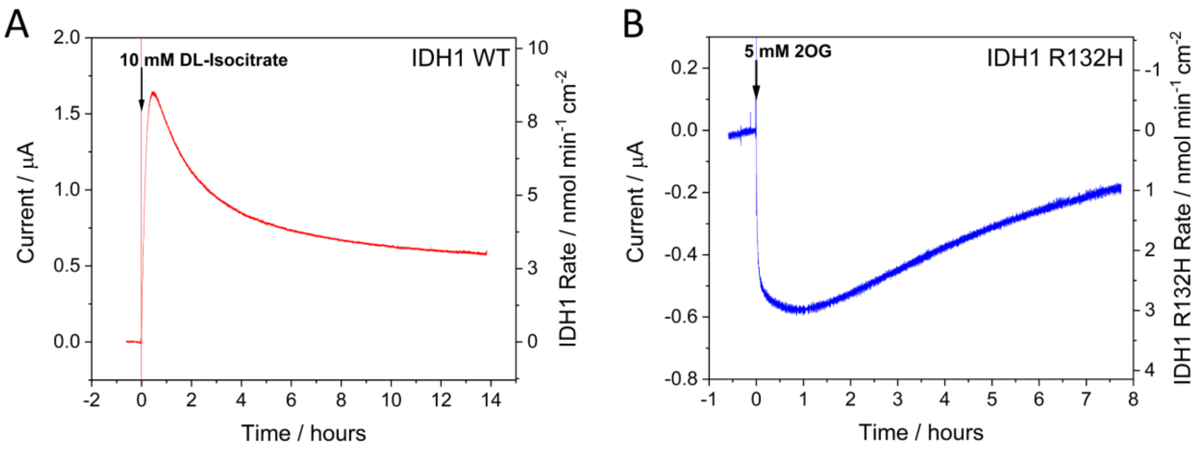

Supporting Figure 7. Electrochemical experiments showing the activity of (A) wild-type IDH1 and (B) IDH1 R132H enzyme upon injection of substrate without any added NADP(H). Conditions (A and B): (FNR+E2)@ITO/PGE electrode, area 0.06 cm^2, temperature 25 °C, volume 4 mL, pH = 8 (100 mM HEPES, 10 mM $MgCl_2$, 1000 rpm. (A): potential *E* (vs SHE) = +0.2 V, molar loading ratio: FNR:IDH1; 2:1. (B): potential *E* (vs SHE) = –0.5 V, molar loading ratio: FNR:IDH1 R132H; 1:2.

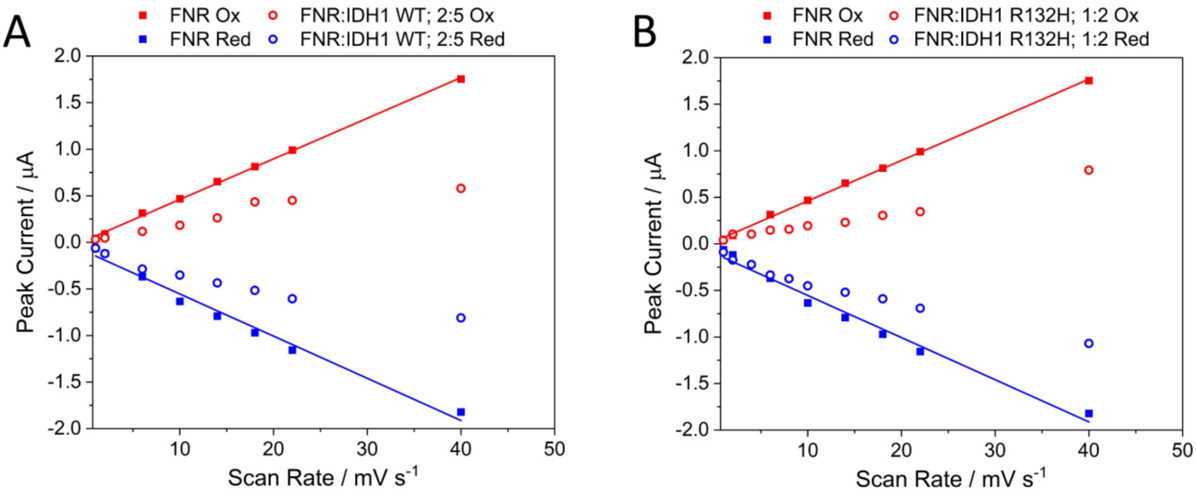

Supporting Figure 8. Cyclic voltammetry peak current values plotted against scan rate for an FNR only electrode (solid squares) and electrodes coloaded with both FNR and IDH1 (open circles). The plots show that when FNR is loaded alone, the peak current is linear with scan rate as expected for an adsorbed electroactive protein. However, when FNR is coloaded with wild-type IDH1 (A) or IDH1 R132H (B), the peak current is not linear with scan rate indicating the presence of an electroactive species that is not adsorbed.

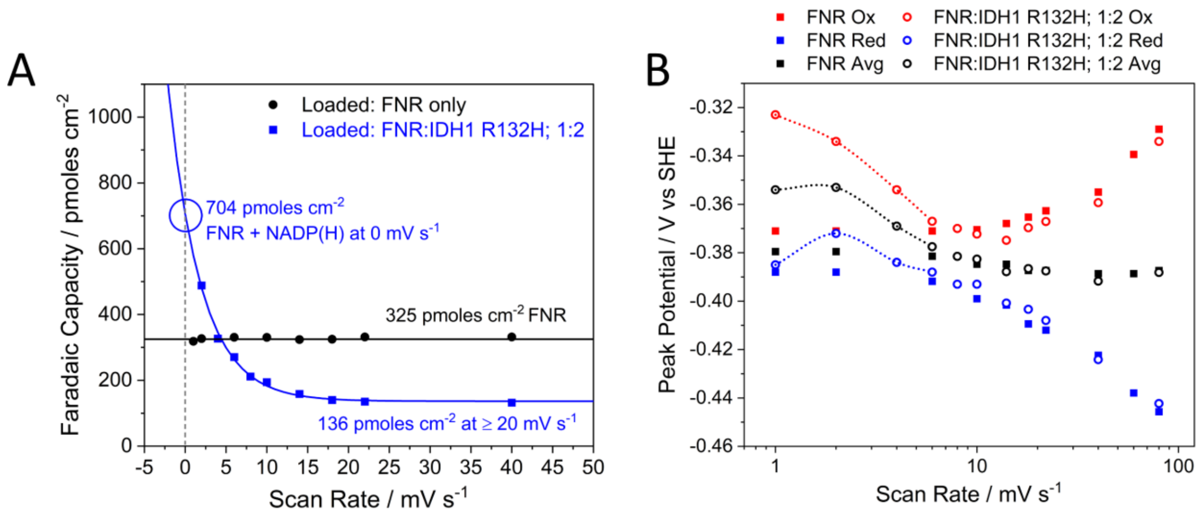

Supporting Figure 9. Scan rate-dependent Faradaic capacity (A) and trumpet plot (B) from thin-film voltammetry experiments using an electrode coloaded with FNR and IDH1 R132H compared to an FNR-only electrode. At low scan rates, the NADP(H) carried into the electrode nanopores with IDH1 R132H can be detected; peaks collapse to the FNR-only signal at higher scan rates. (A) Scan rate dependent coverage plot fitted with an asymptotic exponential equation to allow extrapolation to 0 mV/s. (B) Trumpet plot showing the changes in oxidation and reduction peak potentials as a function of scan rate (see trend lines). Conditions: stationary (FNR+IDH1 R132H)@ITO/PGE electrode (except for FNR-only data, which did not contain IDH1), electrode area 0.06 cm^2, temperature 25 °C, volume 4 mL, pH = 8 (100 mM HEPES), enzyme loading ratios (molar): FNR/IDH1 R132H; 1/2.

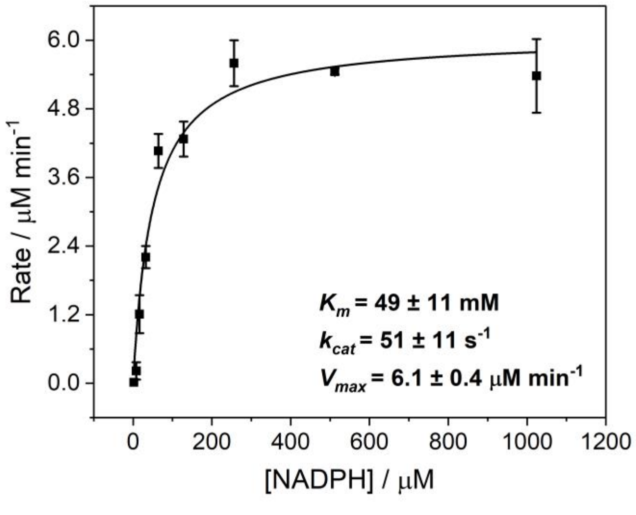

Supporting Figure 10. Michaelis-Menten fit of FNR-catalyzed NADPH oxidation in dilute solution using benzyl viologen as an electron sink. Conditions: 2 nM FNR, 100 mM HEPES (pH = 8), 5 mM benzyl viologen, 26 °C, [NADPH] was varied, 1 mL reaction volume. Reactions were carried out in duplicate. See **Materials and Methods** for further details.

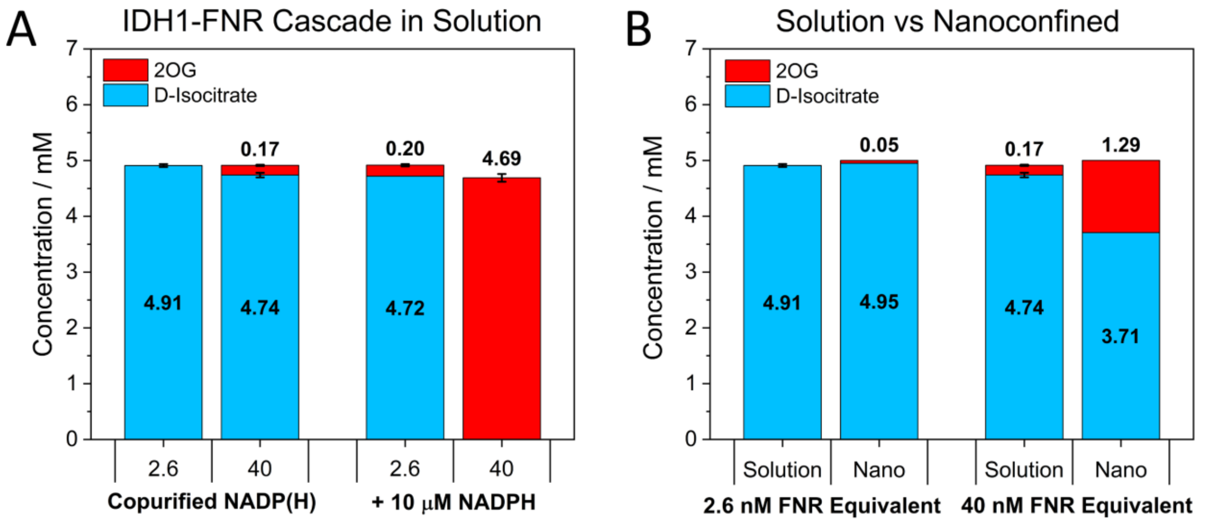

Supporting Figure 11. Comparison of the IDH1-FNR cascade in solution vs under nanoconfinement. (A) Analysis (by ^{1}H NMR after heat quenching) showing the solution-based conversion of isocitrate to 2OG after 12 hours. The numbers under each bar in the x-axis denote the concentration (in nM) of FNR used. The molar ratio of FNR:IDH1$_{dimer}$ used was 1:1.04 ([IDH1] = 1.04 x [FNR]). The "Copurified NADP(H)" bars show experiments where no NADP(H) was added (only IDH1-copurified NADP(H) was present); the "+ 10 µM NADPH" bars show positive control experiments where 10 µM NADPH was added. Conditions: 400 µL volume, 100 mM HEPES (pH 8), 10 mM $MgCl_2$, 5 mM D-isocitrate, 25 mM benzyl viologen. All reactions were performed in triplicate with error bars representing the standard deviation. (B) Comparison of the IDH1-FNR cascade in solution vs under nanoconfinement without any added NADP(H). The "Copurified NADP(H)" solution experiments in (A) are compared with equivalent amounts of enzyme normalized to the reaction volume (4 mL) used in the analytical-scale electrochemical experiment from **Supporting Figure 7A** ("2.6 nM FNR Equivalent") and the scaled-up electrochemical experiment in **Figure 3** ("40 nM FNR Equivalent").

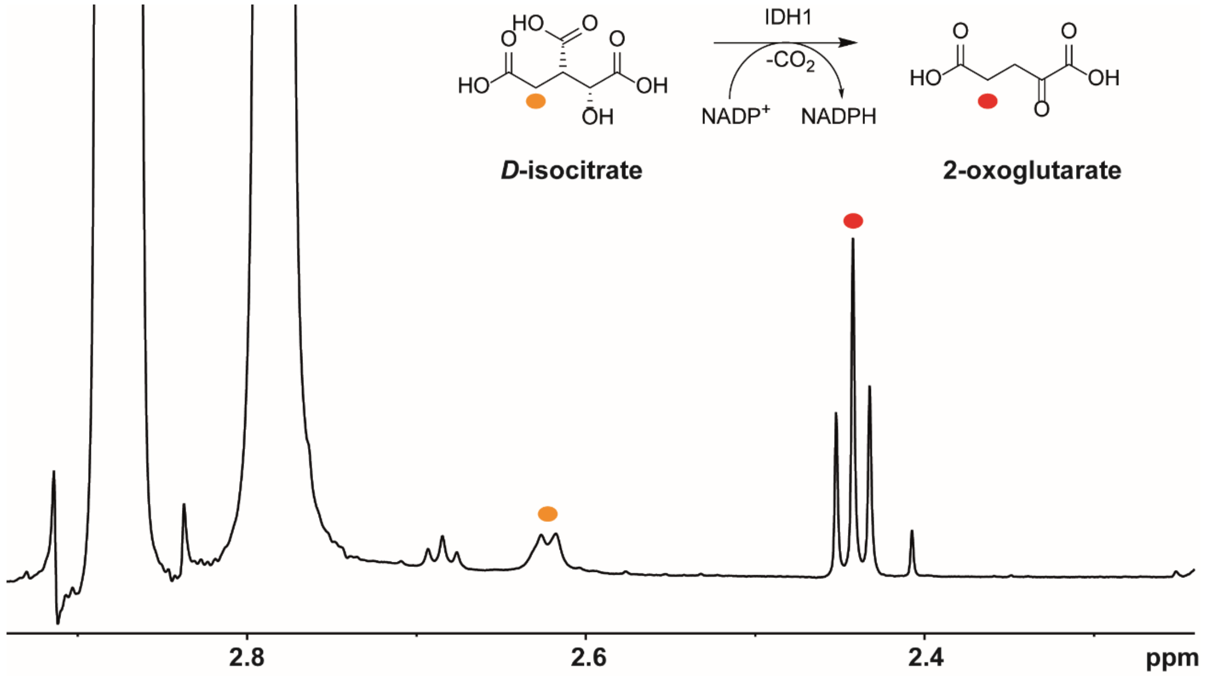

Supporting Figure 12. ^{1}H NMR peak identification for isocitrate and 2OG.

References

1. R. A. Herold, *et al.*, Exploiting Electrode Nanoconfinement to Investigate the Catalytic Properties of Isocitrate Dehydrogenase (IDH1) and a Cancer-Associated Variant. *J. Phys. Chem. Lett.* **12**, 6095–6101 (2021).
2. R. Reinbold, *et al.*, Resistance to the isocitrate dehydrogenase 1 mutant inhibitor ivosidenib can be overcome by alternative dimer-interface binding inhibitors. *Nat. Commun.* **13**, 4785 (2022).
3. R. M. Evans, F. A. Armstrong, "Electrochemistry of Metalloproteins: Protein Film Electrochemistry for the Study of E. coli [NiFe]-Hydrogenase-1" in *Methods in Molecular Biology*, (Humana Press Inc., 2014), pp. 73–94.
4. B. Siritanaratkul, *et al.*, Transfer of photosynthetic NADP+/NADPH recycling activity to a porous metal oxide for highly specific, electrochemically-driven organic synthesis. *Chem. Sci.* **8**, 4579–4586 (2017).
5. S. G. Mayhew, F. Muller, Dimerization of the radical cation of Benzyl Viologen in aqueous solution. *Biochem. Soc. Trans.* **10**, 176–177 (1982).
6. N. R. Bastian, C. J. Kay, M. J. Barber, K. V. Rajagopalan, Spectroscopic studies of the molybdenum-containing dimethyl sulfoxide reductase from Rhodobacter sphaeroides f. sp. denitrificans. *J. Biol. Chem.* **266**, 45–51 (1991).
7. X. Xu, *et al.*, Structures of Human Cytosolic NADP-dependent Isocitrate Dehydrogenase Reveal a Novel Self-regulatory Mechanism of Activity. *J. Biol. Chem.* **279**, 33946–33957 (2004).
8. B. Yang, C. Zhong, Y. Peng, Z. Lai, J. Ding, Molecular mechanisms of "off-on switch" of activities of human IDH1 by tumor-associated mutation R132H. *Cell Res. 2010 2011* **20**, 1188–1200 (2010).
9. J. V. Roman, T. R. Melkonian, N. R. Silvaggi, G. R. Moran, Transient-State Analysis of Human Isocitrate Dehydrogenase I: Accounting for the Interconversion of Active and Non-Active Conformational States. *Biochemistry* **58**, 5366–5380 (2019).
